# Recurrent facial microcystic adnexal carcinoma and hair transplantation on scar site

**DOI:** 10.1002/ccr3.7453

**Published:** 2023-06-08

**Authors:** Amir Mohammad Beyzaee, Mohamad Goldust, Anant Patil, Ghasem Rahmatpour Rokni, Bahare Ghoreishi

**Affiliations:** ^1^ Department of Dermatology Mazandaran University of Medical Sciences Sari Iran; ^2^ Department of Dermatology University Medical Center of the Johannes Gutenberg University Mainz Germany; ^3^ Department of Pharmacology Dr. DY Patil Medical College Navi Mumbai India; ^4^ Department of Dermatology, Faculty of Medicine Mazandaran University of Medical Sciences Sari Iran

**Keywords:** facial tumor, hair transplantation, microcystic adnexal carcinoma, scar

## Abstract

Microcystic adnexal carcinoma (MAC) is a rare kind of cutaneous neoplasm with a very aggressive local infiltration that destructs the affected tissues. Its rate of recurrence is high and it mostly involves the face and scalp regions and most of the patients get affected in the fourth or fifth decades of their life. Here in, we report a 61‐year‐old woman with a right‐sided eyebrow MAC lesion with recurrency. Total excisional surgery was performed. A‐T Flap surgery was applied on the involved area, and after a 2‐year period of follow‐up, with no recurrency, hair transplantation with follicular unit transplantation method was successfully performed on the scarred area. Although microcystic adnexal carcinoma is an uncommon neoplasm; dermatologists and ophthalmologists should consider it as a differential diagnosis, due to its aggressive local infiltration. Complete surgical excision and long‐term follow‐up must be applied to manage the disease. Also, hair transplantation with follicular unit transplantation technique can be considered as a beneficial method for treating scars resulted from MAC excisional surgery.

## 1 INTRODUCTION

Microcystic adnexal carcinoma (MAC), first described by Goldstein in 1982,^1^ is a rare kind of cutaneous neoplasm with a very aggressive local infiltration that destructs the affected tissues.^2^ MAC recurs frequently and the ratio of male and female patients affected by, is almost equal. Head and neck are the most involved regions.

Its age range is 11–83 years (mostly appears in the 40–50) and is described as a firm, colored lesion (papule, nodule, or plaque) which grows slowly.^2^, ^3^, ^4^, ^5^, ^6^

Pain, ignition, and decreased sensation or paresthesia (which is a sign of perineural invasion) are considered as the most common symptoms of MAC.^2^, ^7^, ^8^, ^9^, ^10^

Although MAC is not a metastatic tumor^5^; it infiltrates, shelves, or skates among the facial tissues (including muscle, perichondrium, periosteum, and galea) and aggressively invades the affected region^8^ and spreads far beyond the visible margins.^11^

According to its clinical and histological features, it can easily be misdiagnosed or confused with other cutaneous neoplasms like basal cell carcinoma, squamous cell carcinoma, desmoplastic trichoepithelioma, syringoma, and trichoadenoma.^12^

The correct diagnosis of MAC is very vital because of its aggressive behavior and high recurrence rate. Here in, we report a case of recurrent MAC and discuss about possible treatments.

## 2 CASE PRESENTATION

A 61‐year‐old woman referred to our dermatology clinic with a recurrent lesion on her right eyebrow since the last 3 years. The lesion had no pain and had been enlarging slowly during these years. She declared avoidance of excessive sun exposure or radiotherapy on the head or neck region. Excisional surgery had been performed twice on the lesion, and it has been recurred again now.

**FIGURE 1:**
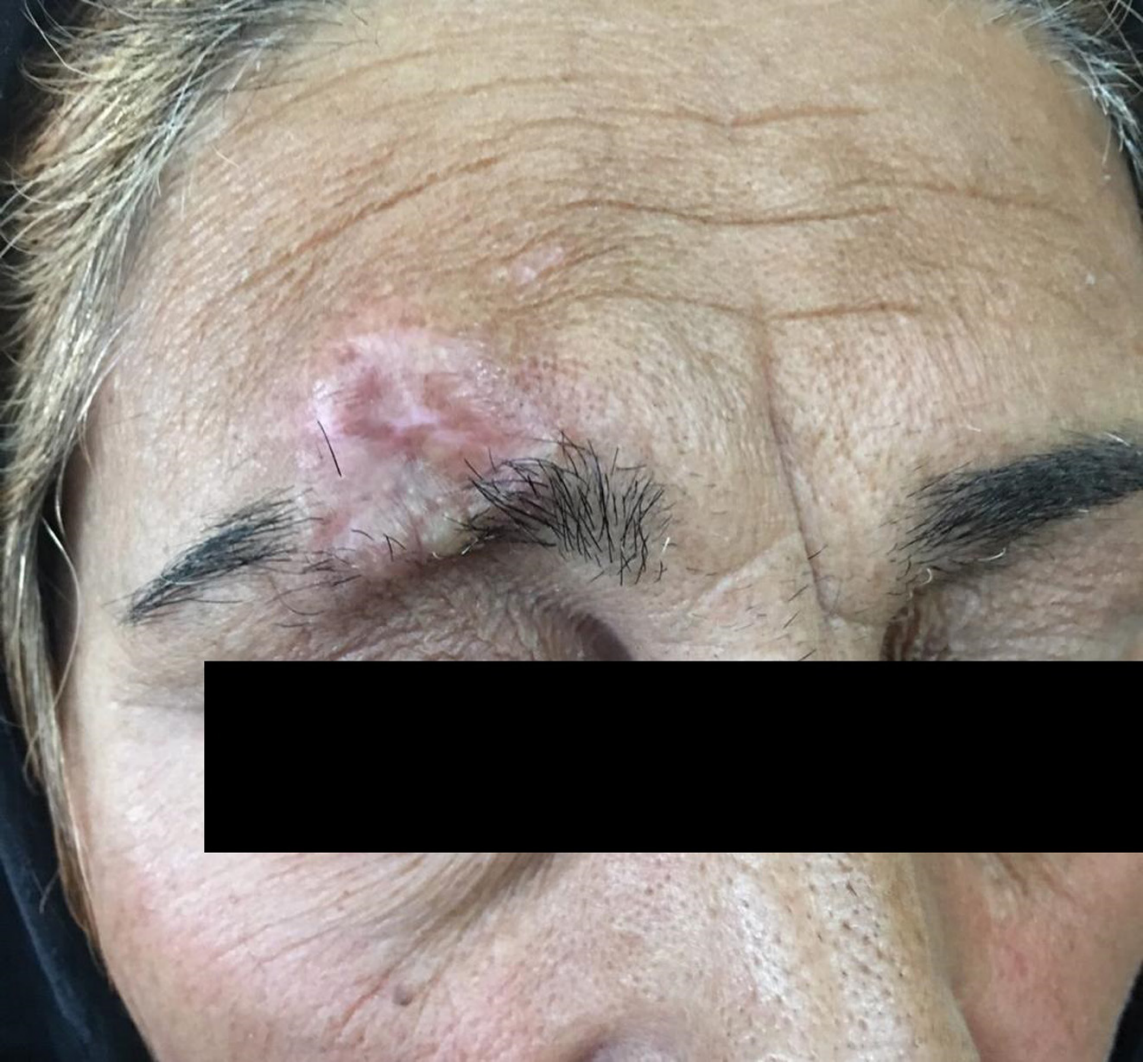
The lesion before surgery.

On the examination, there was a round and elevated lesion with 25 × 30 mm diameter on the right eyebrow. The surface of the lesion was nodular and indurated (Figure 1). The lesion was firm and had no attachment to the underlying periosteum; no skin ulceration or regional lymphadenopathy was seen. The patient declared no tenderness.

After excision biopsy, pathology reported adnexal neoplasm consistent with microcystic adnexal carcinoma.

**FIGURE 2:**
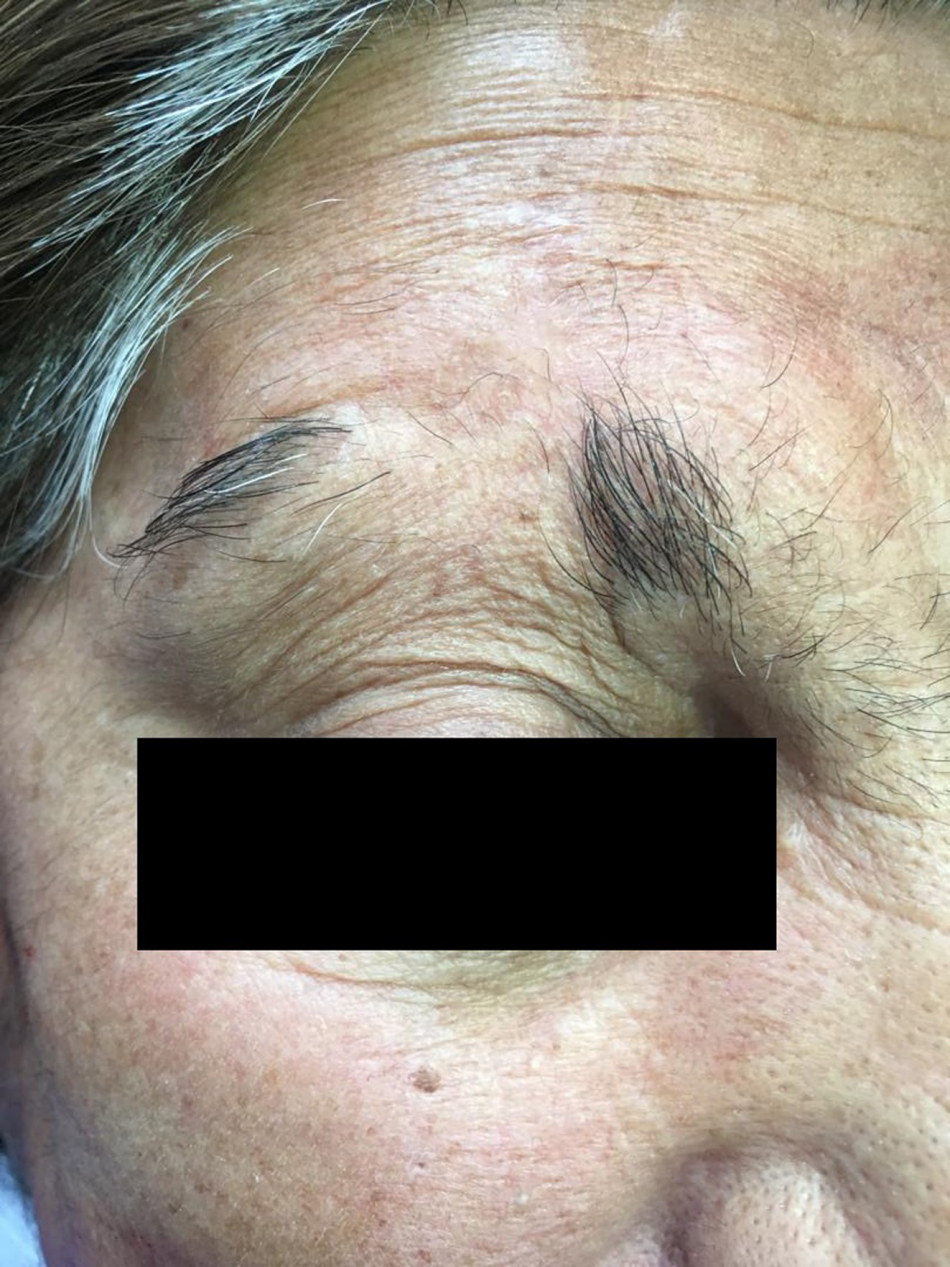
The lesion after excisional surgery (A‐T Flap).

Total excisional surgery was performed. Finally, A‐T Flap surgery was applied on the involved area (Figure 2).

The physicians offered her to do radiotherapy, but she refused.

**FIGURE 3:**
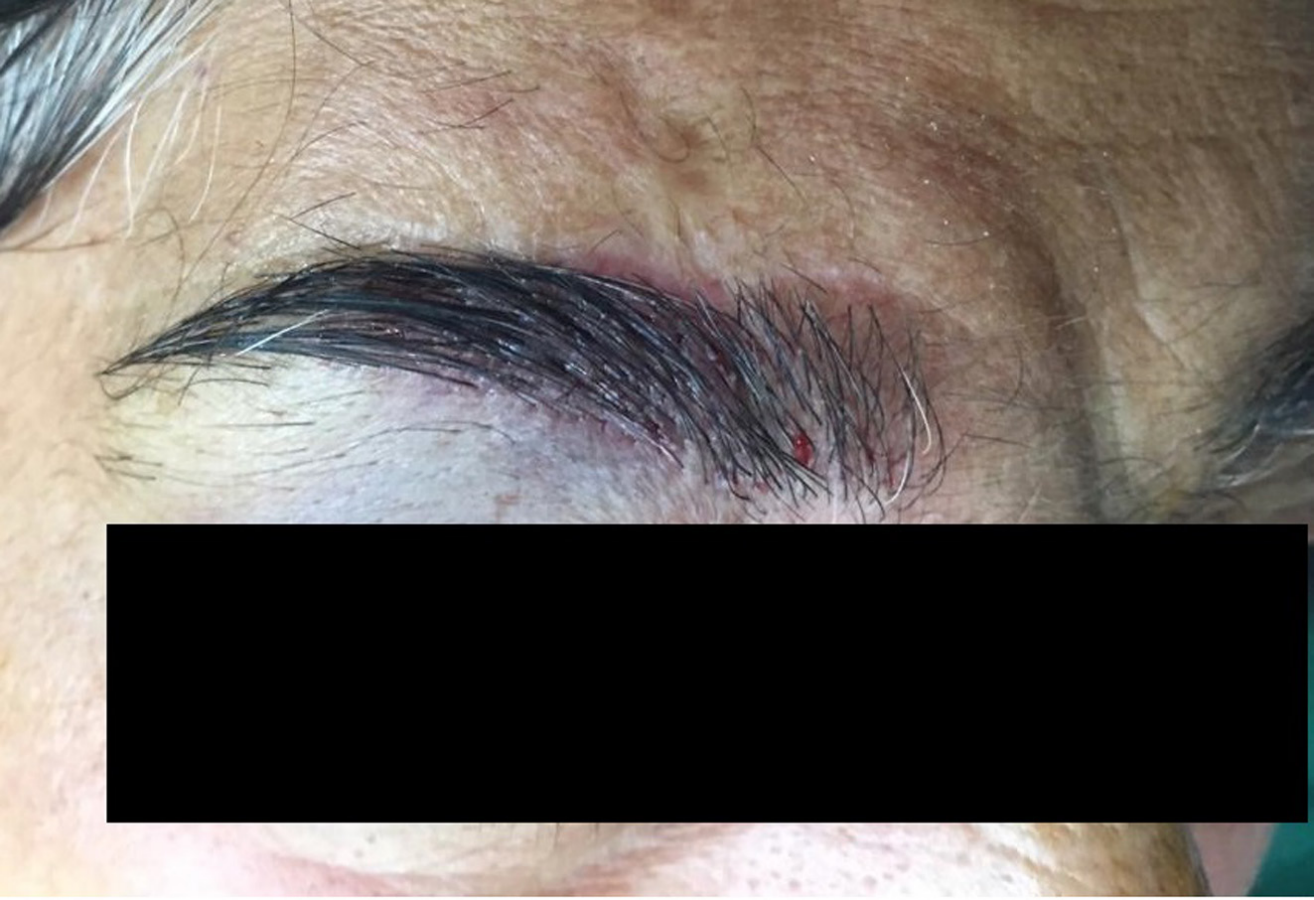
The lesion after 6 month of hair transplantation with follicular unit transplantation technique.

After a 2‐year period of follow‐up, with no recurrency, hair transplantation with follicular unit transplantation method was successfully performed on the scarred area (Figure 3).

## 3 DISCUSSION

MAC mostly occurs in the centrofacial region, in which lips include about 50% of cases.^8^

The other most common involved sites are periocular area, nose, nasolabial fold, cheek, scalp, and chin. Although MAC is mostly considered as a neoplasm of the head and neck region; the axilla, antecubital fossa, buttocks, foot, and chest are the other affected areas which have been reported in the literature.^2^, ^4^, ^12^, ^13^

MAC is assumed to be a non‐metastatic tumor; however, Yugueros et al. reported 55 patients affected to sweat gland carcinomas, in which, 17 of them declared a history of MAC which was treated by excisional surgery and radiotherapy.^14^ Also Lei et al. reported lymph node involvement in the contralateral region of neck in a patient with MAC, which had performed radiation and surgery as treatment 22 years earlier. They proposed that it is more possible to be the result of direct extension via perineural path rather than lymphatic spread.^4^, ^15^ The supraorbital nerve can be invaded by the lesions presenting in periorbial area, particularly on the eyelid or eyebrow.

Although no definitive cause has been found yet, some studies have reported radiation therapy, chronic sun exposure, and immunocompromised status as predisposing factors for MAC.^2^, ^3^, ^8^, ^16^

History of prior radiation has been reported in 8%–12% of the cases^17^, ^18^, ^19^; also lesions in atypical sites, including the neck, have been seen in patients with a history of radiation on the affected area.^20^, ^21^

It is proposed that taking immunosuppressive drugs may develop MAC lesions.^22^ A chronic lymphocytic leukemia (CLL) patient with scalp MAC was reported by Carroll et al.^23^; as it has been reported in immunocompromised patients several times.^22^, ^23^, ^24^

In order to have a successful treatment, an accurate diagnosis is necessary. Clement et al. found that MAC can be easily misdiagnosed.^25^ As mentioned before, the clinical presentation of MAC could be mistaken for basal cell carcinoma (BCC), squamous cell carcinoma, cyst or scar, particularly when a biopsy is not representative of the entire lesion. Failure to obtain a proper biopsy sample could also result misdiagnosis, due to the infiltrative nature of the lesion.

We cannot evaluate the treatment options of MAC properly, due to the rarity of the disease and lack of studies with long‐term follow‐up of the patients in the literature. As recurrence of MAC is not predictable, excision surgery with clear margins and minimum pathologic atypia can aid to decrease the rate of recurrence.^2^

The most common utilized treatment modalities for MAC include wide local excisional surgery (WLE), Mohs micrographic surgery (MMS), and radiotherapy. Recurrence has been reported in using every type of treatment modalities above, even in patients with clear tumor margins.^3^, ^8^, ^15^, ^26^

Regardless of the method chosen, complete removal of the tumor on its first occurrence is ideal as recurrent lesions are much more difficult to manage.

MMS offers the highest probability of clear surgical margins as it can detect subclinical extension while providing maximum tissue sparing.^2^, ^27^

Determining true margin status in the subset of MAC patients with perineural invasion (PNI) treated with surgical techniques can be problematic. Cutaneous carcinoma with PNI manifestation, behaves aggressively and increased rate of recurrence with wide extension have been observed. It has been reported in 17.5%–59% of MAC patients.^27^

WLE is another alternative, in which margin detection depends mainly on the histologic technique hired by the pathologist.

It is strongly recommended to perform complete excisional surgery with clear margins with long‐term follow‐up, because of high rate of recurrences; as it can recur even several years after the surgery.^2^, ^28^ Also we should take surgical margins wider than clinical margins due to the infiltrative growth pattern of the tumor. Recurrence rate of WLE is about 17%–60%, as reported in studies.^2^, ^8^, ^29^, ^30^

It is still challenging to define the role for definitive or adjunctive radiotherapy in, because of the rarity of disease and the uncommon use of radiotherapy for managing the disease. The majority of studies reported unclear details about hired techniques of radiotherapy for MAC lesions. Differences in radiotherapy techniques and sequencing also make it difficult to draw conclusions. There is evidence that radiotherapy can transform the MAC lesion into a new highly aggressive form, according to its clinical and histological features.^31^, ^32^

MAC has also been reported as a radiogenic secondary malignancy.^33^ Sixteen of 84 published MAC cases in the literature, have declared a history of prior exposure to radiations, with 19.05% incidence.^8^, ^17^, ^19^, ^28^, ^31^, ^34^, ^35^, ^36^, ^37^, ^38^, ^39^, ^40^, ^41^, ^42^, ^43^, ^44^

As the rate of MAC recurrence after monotherapy with radiotherapy is high,^1^, ^8^, ^45^ it is not commonly suggested to use radiotherapy as definitive monotherapy for patients with MAC. But as adjuvant therapy, it may be useful in subset of patients who have undergone WLE or MMS without achieving a tumor free margin and are at high risk of recurrence. Also it can be considered as treatment in patients who avoid to perform WLE or MMS, because of the disfiguring facial defects of surgery. Elderly patients may also be a good candidate for radiotherapy, particularly in those whose comorbidities make them poor surgical candidates.

The knowledge about hair transplantation is growing every day. In 1959, Dr. Orentreich developed it for the first time. He applied it as a therapeutic procedure on a patient suffering from androgenic alopecia.^46^

Hair transplantation is used for covering the features of hair loss and it has become one of the main popular procedures in cosmetic field (particularly scars) now with satisfactory results.^47^

Follicular unit transplantation (FUT) and follicular unit extraction (FUE) are common techniques hired for hair transplantation. In FUT technique, a band‐shaped tissue of occipital region is used as donor, which results a linear scar. To avoid its complications, including scarring, FUE technique was introduced, in which, small units of hair follicles are harvested.^47^

Osman et al. performed hair transplantation on the cleft lip area scar. They hired fat grafting procedure on 20 patients suffering from scar and alopecia. Then hair transplantation was performed 3 months after fat injection. After a one‐year period of follow‐up, patients declared noticeable high level of satisfaction.^48^

Also Soyeon et al. evaluated the results of hair transplantation for scar management in 25 cases (of 23 patients), in which burns, operation, and trauma resulted scar with hair loss on the scalp and the face (eyebrow, lip, and eyelid). After a 6 months period of follow‐up, satisfactory report from hair follicle transplantation procedure was as excellent (44.4%), good (38.9%), fair (11.1%), and poor (5.6%).^49^

Transplanting the hair follicles on a tissue with scar is so difficult, due to the poor blood supplement and tenacity of the scar tissue. Also, a higher rate of success and favorable results is seen in patients with burned scar rather than incision scars, due to the deeper depth of incision scars.^49^

So, we should always warn the patients that choosing hair follicle transplantation procedure for scar management, can be accompanied by secondary (or more) operations, for better cosmetic result.

## 4 CONCLUSION

Although microcystic adnexal carcinoma is not a common tumor; dermatologists and ophthalmologists should consider it as a differential diagnosis, due to its aggressive local infiltration. Complete surgical excision and long‐term follow‐up must be applied to manage the disease.

Also, hair transplantation with follicular unit transplantation technique can be considered as a beneficial method for treating scars resulted from MAC excisional surgery.

## AUTHOR CONTRIBUTIONS

**Amir Mohammad Beyzaee:** Conceptualization; data curation; formal analysis; investigation; resources; software; writing – original draft; writing – review and editing. **Mohamad Goldust:** Conceptualization; supervision. **Anant Patil:** Methodology; validation. **Ghasem Rahmatpour Rokni:** Conceptualization; funding acquisition; supervision. **Bahare Ghoreishi:** Data curation; visualization.

## FUNDING INFORMATION

The funding was provided by Ghasem Rahmatpour Rokni and Amir Mohammad Beyzaee.

## CONFLICT OF INTEREST STATEMENT

We declare that none of the authors have any conflict of interest.

## CONSENT

Written informed consent was obtained from the patient to publish this report in accordance with the journal's patient consent policy.

## DECLARATIONS

We declare that the authors listed on the manuscript are not employed by a government agency that has a primary function other than research and/or education. We declare that any of the authors are not submitting this manuscript as an official representative or on behalf of the government.

## Data Availability

The data are available on request.
